# Highly Selective End-Tagged Antimicrobial Peptides Derived from PRELP

**DOI:** 10.1371/journal.pone.0016400

**Published:** 2011-01-27

**Authors:** Martin Malmsten, Gopinath Kasetty, Mukesh Pasupuleti, Jan Alenfall, Artur Schmidtchen

**Affiliations:** 1 Department of Pharmacy, Uppsala University, Uppsala, Sweden; 2 Division of Dermatology and Venereology, Department of Clinical Sciences, Lund University, Lund, Sweden; 3 Dermagen AB, Lund, Sweden; Institut de Pharmacologie et de Biologie Structurale, France

## Abstract

**Background:**

Antimicrobial peptides (AMPs) are receiving increasing attention due to resistance development against conventional antibiotics. *Pseudomonas aeruginosa* and *Staphylococcus aureus* are two major pathogens involved in an array of infections such as ocular infections, cystic fibrosis, wound and post-surgery infections, and sepsis. The goal of the study was to design novel AMPs against these pathogens.

**Methodology and Principal Findings:**

Antibacterial activity was determined by radial diffusion, viable count, and minimal inhibitory concentration assays, while toxicity was evaluated by hemolysis and effects on human epithelial cells. Liposome and fluorescence studies provided mechanistic information. Protease sensitivity was evaluated after subjection to human leukocyte elastase, staphylococcal aureolysin and V8 proteinase, as well as *P. aeruginosa* elastase. Highly active peptides were evaluated in *ex vivo* skin infection models. C-terminal end-tagging by W and F amino acid residues increased antimicrobial potency of the peptide sequences GRRPRPRPRP and RRPRPRPRP, derived from proline arginine-rich and leucine-rich repeat protein (PRELP). The optimized peptides were antimicrobial against a range of Gram-positive *S. aureus* and Gram-negative *P. aeruginosa* clinical isolates, also in the presence of human plasma and blood. Simultaneously, they showed low toxicity against mammalian cells. Particularly W-tagged peptides displayed stability against *P. aeruginosa* elastase, and *S. aureus* V8 proteinase and aureolysin, and the peptide RRPRPRPRPWWWW-NH_2_ was effective against various “superbugs” including vancomycin-resistant enterococci, multi-drug resistant *P. aeruginosa*, and methicillin-resistant *S. aureus*, as well as demonstrated efficiency in an *ex vivo* skin wound model of *S. aureus* and *P. aeruginosa* infection.

**Conclusions/Significance:**

Hydrophobic C-terminal end-tagging of the cationic sequence RRPRPRPRP generates highly selective AMPs with potent activity against multiresistant bacteria and efficiency in *ex vivo* wound infection models. A precise “tuning” of toxicity and proteolytic stability may be achieved by changing tag-length and adding W- or F-amino acid tags.

## Introduction

In order to control microbial flora, humans are armoured with a rapidly acting antimicrobial system based on short cationic and amphiphilic antimicrobial peptides (AMP), which constitute an integral part of innate immunity. At present, there are approximately 1600 identified AMPs (see http://aps.unmc.edu/AP/main.php). Linear AMPs, such as the cathelicidin LL-37, but also magainin-2, PGLa, and pleurocidin, adopt highly ordered amphipathic helices in phospholipid environments and upon bacterial binding [1], [2], [3], [4], [5], [6], [7]. Other peptides, such as α- and β-defensins, comprise amphipathic cysteine-linked antiparallel β-sheets [8], [9]. AMPs may also, however, be found among peptides not displaying such ordered structures as long as these are characterized by an over-representation of certain amino acids, such as histidine (e.g., histatins), or arginine (e.g., PR39) [1], [2], [3], [4], [10]. AMP function has been thought to involve direct binding to the lipid bilayer, and the interaction with bacterial membranes is a prerequisite for AMP function. However, the modes of action of AMPs on their target bacteria are complex, and can be divided into membrane disruptive and non-membrane disruptive [3], [11], [12], [13].

It has become increasingly clear that AMPs belong to a multifunctional group of molecules that interact not only with microbes, but also with negatively charged glycosaminoglycans (such as heparin), biomembranes, and various cell receptors. Apart from their antibacterial actions, biological effects exerted by AMPs include growth stimulus and angiogenesis, protease inhibition, anti-angiogenesis, and chemotaxis [14], [15], [16]. Conversely, cationic peptide motifs from proteins not previously considered as AMPs have been shown to exert antimicrobial activities. For example, complement C3 [17], kininogen [18], [19], heparin-binding protein [20], heparin-binding epidermal growth factor and other growth factors [21], matrix proteins such as laminin, fibronectin and proline arginine-rich end leucine-rich repeat protein (PRELP)[22], prions [23], β2-glycoprotein [24], histidine-rich glycoprotein [25], thrombin [26], and tissue factor pathway inhibitor [27], may, either as holoproteins or smaller peptide derivatives or fragments thereof, also exert antimicrobial activities i*n vitro*, and in several cases, *in vivo* [18], [19], [25]. In general, these findings are compatible with the observation that consensus heparin-binding peptide sequences (Cardin and Weintraub motifs) XBBBXXBX or XBBXBX (where X represents hydrophobic or uncharged amino acids, and B represents basic amino acids), represented by multiples of the motifs ARKKAAKA or AKKARA [28], are antibacterial [29] and specifically interact with membranes [30].

Infectious diseases account for millions of deaths worldwide each year and incur tremendous health care costs. The disease spectrum is broad and includes acute disease, such as acute local or invasive infections, including sepsis, having a direct association to a given pathogen, as well as chronic diseases, where microbes often cause a long-standing inflammatory state. Pathogens such as *Staphylococcus aureus* and *Pseudomonas aeruginosa* cause, and/or aggravate, a spectrum of diseases including bacterial conjunctivitis and keratitis, otitis, postoperative and burn wound infections, chronic leg ulcers, pneumonia, and cystic fibrosis. Community-acquired MRSA has now emerged as an epidemic that is responsible for rapidly progressive, fatal diseases including necrotizing pneumonia, severe sepsis, and necrotizing fasciitis [31]. Concerning streptococci, strains of *S. pyogenes* resistant to macrolide antibiotics have emerged, however all strains still remain uniformly sensitive to penicillin. In addition, enterococci, leading causes of nosocomial bacteremia, surgical wound infection, and urinary tract infection, are becoming intrinsically resistant to many antibiotics [32]. *P. aeruginosa* is emerging, particularly in critically ill patients that require intensive care and are treated with multiple antibiotic agents (see http://www.cdc.gov for further information). Therefore, multi-drug resistant *P. aeruginosa* infections are associated with severe adverse clinical outcomes [33]. As mentioned above, all these bacteria may complicate wounds. In addition, wound infection is one of the most common surgical complications, leading to significant mortality and morbidity. Surgical site infections are the most common form of hospital-acquired infections for surgical patients and occur in between 10-38% (UK and US respectively) of patients. These infections, which occur in 15% of elective surgical patients and approximately 30% of surgical patients whose procedure was classed as contaminated or “dirty”, delay wound healing, prolong hospital stay, cause unnecessary pain and also, increase the risk for invasive infections and sepsis [34].

Considering the increasing resistance problems against conventional antibiotics, AMPs have recently emerged as potential therapeutic candidates in the above conditions [35]. For example, the indolicidin-derived peptide omiganan is currently being evaluated in Phase III clinical trials for treatment of catheter-related infections [36]. However, the use of AMPs in this context is challenging, as bacteria are able to excrete proteolytic enzymes [37], [38], and in the case of *P. aeruginosa* also AMP-scavenging exopolysaccharides, as a defense against AMPs. Furthermore, *S. aureus* displays an impressive number of resistance mechanisms, including net charge alterations [39], [40]. Thus, the teichoic acid polymers found in the cell wall of this bacterium, as well as in those of other Gram-positives, normally having strong anionic properties mediated by phosphate groups of the glycerolphosphate repeating units, can be modified by D-alanine residues with free amino groups. Analogously, the major (and negatively charged) lipid phosphatidylglycerol is modified into a net positive charge by addition of L-lysine [3], [41]. Ideally, therefore, AMPs should display high bactericidal potency and protease stability, but low toxicity against mammalian cells. Various strategies, such as use of combinational library approaches [42], stereoisomers composed of D-amino acids [43] or cyclic D,L-α-peptides [44], high-throughput based screening assays [45], [46], quantitative structure-activity relationship (QSAR) approaches [35], [45], [47], [48], and identification of endogenous peptides [22], [26], [49], [50], [51], [52], are currently employed for identifying selective and therapeutically interesting AMPs [53], [54]. Utilization of endogenous antimicrobial peptide sequences could constitute an attractive alternative strategy in order to develop novel antiinfectives, and as mentioned above, a PRELP-derived peptide, QPTRRPRPGTGPGRRPRPRPRP, was previously found to exert antimicrobial effects against both *P. aeruginosa* and *S. aureus* [22] **.** Analysis by fluorescence microscopy demonstrated that QPT22 bound to bacterial membranes and induced membrane leakage of liposomes. Furthermore, the peptide displayed no hemolytic activity, nor did it exert membrane permeabilising effects on human epithelial cells. In a parallel line of research, we previously identified end-tagging of AMPs with hydrophobic amino acid stretches as an effective approach to achieve high adsorption of partially submerged, highly charged AMPs [55], [56], [57]. Given this, and the abovementioned ability of *S. aureus* to reduce its surface charge density, hydrophobic end-tagging of AMPs with hydrophobic amino acid stretches is an interesting way to improve bactericidal potency of AMPs. Particularly for short, highly positively charged, and hydrophilic peptides, this facilitates the design of potent, but selective, AMPs. Previous physico-chemical investigations, involving studies on peptide adsorption at supported lipid bilayers, peptide-induced liposome rupture, as well as LPS-binding experiments, circular dichroism experiments on peptide conformation, and studies on bacterial wall rupture, demonstrated that the end-tagged peptides reach their potency and salt resistance through the hydrophobic end-tags, promoting peptide adsorption at phospholipid membranes. The selectivity between bacteria and eukaryotic cells could also be explained on a mechanistic level, and due to the lower charge density of eukaryotic cell membrane, combined with the presence of cholesterol in the latter [55], [56], [57]. Based on these results, and considering the promising results with the PRELP-derived AMP mentioned above [22], we selected the peptides GRRPRPRPRP (GRR10) and RRPRPRPRP (RRP9) (derived from the C-terminal part of QPTRRPRPGTGPGRRPRPRPRP) as templates for C-terminal tagging hydrophobic amino acid stretches in order to generate novel effective AMPs.

Herein the present report, we demonstrate that tagging these PRELP-derived AMPs with W and F amino acid residues may be employed to reach very high bactericidal potency against various important Gram-negative and Gram-positive pathogens at maintained limited toxicity. Furthermore, toxicity as well as proteolytic stability may be selectively tuned. In addition, by varying tag length and composition, we demonstrate that incorporation of W-stretches, in contrast to F, yields good stability against human elastase, as well as *S. aureus* and *P. aeruginosa* proteases. This is an important aspect for the therapeutic use of AMPs in environments containing high proteolytic activity, such as those occurring during inflammation and infection.

## Materials and Methods

### Ethics statement

The use of human blood was approved by the Ethics Committee at Lund University (657-2008). Written informed consent was obtained from the donors.

### Peptides

Peptides used in this work (Table 1) were synthesized by Biopeptide Co., San Diego, USA, with the exception of LL-37 (LLGDFFRKSKEKIGKEFKRIVQRIKDFLRNLVPRTES), which was obtained from Innovagen AB, Lund, Sweden. The purity (>95%) of these peptides was confirmed by mass spectral analysis (MALDI-ToF Voyager), provided by the suppliers. Peptides were diluted in H_2_0 (5 mM stock), and stored at –20°C, until used. This stock solution was used for the subsequent experiments.

**Table 1:** Characteristics of peptides used in the study.

Sequence	HydrophobicityKyte & Dolittle	HydrophobicityCCS scale	net charge
GRRPRPRPRP-COOH	−2.93	−5.32	+5
GRRPRPRPRPWWW-COOH	−2.46	−1.85	+5
GRRPRPRPRPWWWW-COOH	−2.35	−1.02	+5
GRRPRPRPRPWWWWW-COOH	−2.25	−0.31	+5
GRRPRPRPRP-NH_2_	−2.93	−5.32	+6
GRRPRPRPRPWWW-NH_2_	−2.46	−1.85	+6
GRRPRPRPRPWWWW-NH_2_	−2.35	−1.02	+6
GRRPRPRPRPFFF-COOH	−1.6	−1.78	+5
GRRPRPRPRPFFFF-COOH	−1.29	−0.94	+5
GRRPRPRPRPFFFFF-COOH	−1.02	−0.21	+5
GRRPRPRPRPFFF-NH_2_	−1.6	−1.78	+6
GRRPRPRPRPFFFF-NH_2_	−1.29	−0.94	+6
GRRPRPRPRPFFFFF-NH_2_	−1.02	−0.21	+6
RRPRPRPRPWWW-NH_2_	−2.63	−1.8	+6
RRPRPRPRPWWWW-NH_2_	−2.5	−0.92	+6
RRPRPRPRPFFFFF-COOH	−1.06	−0.05	+5
RRPRPRPRPFFF-NH_2_	−1.7	−1.73	+6
RRPRPRPRPFFFF-NH_2_	−1.36	−0.83	+6
RRPRPRPRPFFFFF-NH_2_	−1.06	−0.05	+6
ILRWPWWPWRRK-NH_2_	−1.32	1.4	+5
LLGDFFRKSKEKIGKEFKRIVQRIKDFLRNLVPRTES-COOH	−0.72	−1.84	+6

### Microorganisms

*Escherichia coli* ATCC 25922, *Staphylococcus aureus* ATCC 29213, and *Pseudomonas aeruginosa* ATCC 27853, as well as the other clinical isolates, were obtained from the Department of Clinical Bacteriology at Lund University Hospital. Additional isolates presented in Table 2 were maintained and tested at Quotient Bioresearch, Cardiff, United Kingdom.

**Table 2:** MIC values for the peptide RRP9W4N against various pathogens, including multiresistant “superbugs”.

Bacterial Strain	Cation-adjusted Mueller-Hinton broth	
	RRP9W4N mg/l	RRP9W4N µM
*Staphylococcus aureus* ATCC 29213 – antibiotic-susceptible type strain	16	8.29
*Staphylococcus aureus* ATCC 43300 – methicillin-resistant type strain	16	8.29
*Staphylococcus aureus* – methicillin-resistant clinical isolate	32	16.57
*Staphylococcus aureus* - multi-drug-resistant clinical isolate	32	16.57
*Staphylococcus aureus* - teicoplanin-intermediate clinical isolate	32	16.57
MU50 *Staphylococcus aureus (*MRSA) – VISA type strain	32	16.57
EMRSA3 *Staphylococcus aureus* (MRSA) – SSCmec type 1	16	8.29
EMRSA16 *Staphylococcus aureus* (MRSA) – SSCmec type 2	32	16.57
EMRSA1 *Staphylococcus aureus* (MRSA) – SSCmec type 3	32	16.57
EMRSA15 *Staphylococcus aureus* (MRSA) – SSCmec type 4	16	8.29
HT2001254 *Staphylococcus aureus* (MRSA) – PVL positive	32	16.57
*Staphylococcus epidermidis* – antibiotic susceptible clinical isolate	8	4.14
*Staphylococcus epidermidis*– methicillin-resistant clinical isolate	8	4.14
*Staphylococcus haemolyticus* – antibiotic susceptible clinical isolate	8	4.14
*Staphylococcus saprophyticus* – antibiotic susceptible clinical isolate	64	33.14
Group C Streptococcus – antibiotic-susceptible clinical isolate	8	4.14
Group G Streptococcus – antibiotic-susceptible clinical isolate	32	16.57
Group G Streptococcus – macrolide-resistant clinical isolate	16	8.29
Group C Streptococcus – macrolide-resistant clinical isolate	8	4.14
*Streptococcus pyogenes* – antibiotic-susceptible clinical isolate	32	16.57
*Streptococcus pyogenes* – Macrolide (M-type) resistance clinical isolate	16	8.29
*Streptococcus pyogenes* – Macrolide (MLS) resistant clinical isolate	16	8.29
*Streptococcus pyogenes* – ATCC 19615	16	8.29
*Escherichia coli* ATCC 25922 - antibiotic-susceptible type strain	16	8.29
*Escherichia coli* ATCC 35218 - β-lactamase positive type strain	16	8.29
*Escherichia coli* - multi-drug resistant clinical isolate	16	8.29
*Escherichia coli* - ESBL - TEM	16	8.29
*Escherichia coli* - ESBL - CTXM	16	8.29
*Escherichia coli* - ESBL - SHV	32	16.57
*Pseudomonas aeruginosa* ATCC 27853 - antibiotic-susceptible type strain	64	33.14
*Pseudomonas aeruginosa* - multi-drug resistant clinical isolate	128	66.29
*Pseudomonas aeruginosa* – Liverpool genotype LES 431	128	66.29

MIC assay was carried out by a microtiter broth dilution method as previously described in the NCSLA guidelines [60] in cation-adjusted Mueller-Hinton broth.

### Radial diffusion assay

Radial diffusion assay was used in order to evaluate antibacterial effects. As previously described [58], [59], bacteria were grown to mid-logarithmic phase in 10 ml of full-strength (3% w/v) trypticase soy broth (TSB) (Becton-Dickinson, Cockeysville, USA). The cells were then washed once with 10 mM Tris, pH 7.4. Subsequently, 4×10^6^ bacterial colony forming units were added to 15 ml of the underlay agarose gel, consisting of 0.03% (w/v) TSB, 1% (w/v) low electroendosmosis type (EEO) agarose (Sigma-Aldrich, St. Louis, USA) and 0.02% (v/v) Tween 20 (Sigma-Aldrich, St. Louis, USA). The underlay was poured into a Ø 144 mm petri dish. After agarose solidification, 4 mm-diameter wells were punched and 6 µl of peptide with required concentration was added to each well. Plates were incubated at 37°C for 3 hours to allow diffusion of the peptides. The underlay gel was then covered with 15 ml of molten overlay (6% TSB and 1% Low-EEO agarose in distilled H_2_O). Antimicrobial activity of a peptide is visualized as a zone of clearing around each well after 18–24 hours of incubation at 37°C. Results given represent mean values from triplicate measurements.

### Viable-count analysis

For additional evaluation of bactericidal effects, *Staphylococcus aureus* ATCC 29213 and *Pseudomonas aeruginosa* ATCC 27853 bacteria (50 µl; 2×10^6^ cfu/ml) were grown to mid-logarithmic phase in Todd-Hewitt (TH) medium. Bacteria were washed and diluted in 10 mM Tris, pH 7.4, 5 mM glucose, 0.15 M NaCl, with 20% human citrate-plasma. After peptide exposure, serial dilutions of the incubation mixture were plated on TH agar, followed by incubation at 37°C overnight and cfu determination. In the experiments using 50% whole blood [26], *S. aureus* ATCC 29213 and *P. aeruginosa* ATCC 27853 bacteria (50 µl; 2×10^8^ cfu/ml) were incubated at 37°C for 1 hour in the presence of peptide at 60 and 120 µM. Serial dilutions of the incubation mixture were plated on TH agar, followed by incubation at 37°C overnight and cfu determination.

### Minimal inhibitory concentration (MIC) determination

In order to determine the minimal inhibitory concentration (MIC) for a given peptide and bacteria, we used a standardized dilution method according to NCSLA guidelines [60]. In brief, fresh overnight colonies were suspended to a turbidity of 0.5 units and further diluted in Mueller-Hinton broth (MH) (Becton Dickinson). For determination of MIC, peptides were dissolved in water at concentration 10 times higher than the required range by serial dilutions from a stock solution. Ten µl of each concentration was added to each corresponding well of a 96-well microtiter plate (polypropylene, Costar Corp.) and 90 µl of bacteria (1×10^5^) in MH medium added. The plate was incubated at 37°C for 16–18 h. MIC was considered as the lowest concentration of peptide where no visual growth of bacteria was detected. Additional MIC determinations presented in Table 2 were performed using cation-adjusted MH broth [60] at Quotient Bioresearch Ltd., Fordham, United Kingdom.

### Protease sensitivity assay

For evaluation of sensitivity to bacterial and endogenous proteases, peptides (1 µg) were incubated at 37°C with *S. aureus* aureolysin (0.1 µg, 25000 units/mg), *S. aureus* V8 proteinase (0.1 µg, 2000 mU), both from BioCol GmbH (Potsdam, Germany), human neutrophil elastase (0.4 µg, 29 units/mg; Calbiochem (La Jolla, USA)) or *P. aeruginosa elastase* (0.1 µg, 261 units/mg) (Calbiochem, La Jolla, USA) in a total volume of 30 µl for 3 hours. The materials were analyzed on 16.5% precast sodium dodecyl sulfate polyacrylamide (SDS-PAGE) Tris-Tricine gels (BioRad, Hercules, USA) and analyzed after staining with Coomassie Blue R-250 **(**Merck, Darmstadt, Germany).

### MTT assay

The MTT assay was utilized in order to analyse cell viability of keratinocytes. Sterile filtered MTT (3-(4,5-dimethylthiazolyl)-2,5-diphenyl-tetrazolium bromide; Sigma-Aldrich, St. Louis, USA) solution (5 mg/ml in PBS) was stored protected from light at −20°C until usage. HaCaT keratinocytes (kindly provided by Dr. Robert Fusenig, Heidelberg University, Heidelberg, Germany, [51]), 3000 cells/well, were seeded in 96 well plates and grown in keratinocyte-SFM/BPE-rEGF medium to confluence as previously described. Keratinocyte-SFM/BPE-rEGF medium alone, or keratinocyte-SFM supplemented with 20% serum, was added, followed by peptide addition to 60 µM. After incubation over night, 20 µl of the MTT solution was added to each well and the plates incubated for 1 h in CO_2_ at 37°C. The MTT- containing medium was then removed by aspiration. In the assay, MTT is modified into a dye, blue formazan, by enzymes associated to metabolic activity. The blue formazan product generated was dissolved by the addition of 100 µl of 100% DMSO per well. The plates were then gently swirled for 10 min at room temperature to dissolve the precipitate. The absorbance was monitored at 550 nm, and results given represent mean values from triplicate measurements.

### Lactate dehydrogenase (LDH) assay

The LDH assay was utilized in order to analyse cell permeation of keratinocytes. HaCaT keratinocytes were grown in 96 well plates (3000 cells/well) in serum-free keratinocyte medium (SFM), supplemented with bovine pituitary extract and recombinant EGF (BPE-rEGF) (Invitrogen, Eugene, USA) to confluency. The medium was then removed, and 100 µl of the peptides investigated (at 60 µM, diluted in SFM/BPE-rEGF or in keratinocyte-SFM supplemented with 20% human serum) added in triplicates to different wells of the plate. The LDH-based TOX-7 kit (Sigma-Aldrich, St. Louis, USA) was used for quantification of LDH release from the cells. Results given represent mean values from triplicate measurements, and are given as fractional LDH release compared to the positive control consisting of 1% Triton X-100 (yielding 100% LDH release).

### Hemolysis assay

For analysis of peptide-induced permeabilization of human erythroytes, EDTA-, or citrate-blood was drawn from healthy volunteers [26], [51], centrifuged at 800 g for 10 min, and plasma and buffy coat were removed. For experiments using EDTA-blood, [51] erythrocytes were washed three times and resuspended to 5% in PBS, pH 7.4. For experiments in 50% blood [26], citrate-blood was diluted (1∶1) with PBS. The cells were then incubated with end-over-end rotation for 1 h at 37°C in the presence of peptides (60 µM). 2% Triton X-100 (Sigma-Aldrich, St. Louis, USA) served as positive control. The samples were then centrifuged at 800 g for 10 min. The absorbance of hemoglobin release was measured at 540 nm and is expressed as % of TritonX-100 induced hemolysis. In the experiments with blood infected by bacteria, citrate-blood was diluted (1∶1) with PBS. The cells were then incubated with end-over-end rotation for 1 h at 37°C in the presence of peptides (60 and 120 µM) and *S. aureus* (2×10^8^ cfu/ml) or *P. aeruginosa* (2×10^8^ cfu/ml) bacteria. For evaluation of hemolysis, samples were then processed as above. Results given represent mean values from triplicate measurements.

### Antibacterial effects *ex vivo*

For evaluating antibacterial effects of AMPs *ex vivo*, a pig skin model was used as previously described [61], but with modifications. Defatted pig hides were first washed with water and then 70% ethanol. They were then destubbled with disposable razors and 8×8 cm pieces were cut, sealed in plastic wrap, and frozen at −20°C. Before use, the skin samples were thawed, and then washed with ethanol (70%) and water. In order to separate the inoculation areas, sterilised tubings (polyethylene, 9.6 m, Nalgene® VWR 228-0170) were cut into ∼10 mm lengths, and glued onto the skin samples (cyanoacrylate glue, Henkel, Düsseldorf, Germany). 6 mm punch biopies were then made, and the epidermal parts removed, leaving a dermal wound. The wounded area was infected by adding 1×10^6^ cfu of an overnight culture of *P. aeruginosa* 15159 (clinical chronic ulcer isolate) or *S. aureus* ATCC 29213 in a total volume of 100 µl of human serum diluted in phosphate buffered saline (50∶50). After an incubation time of 2 hours at 37°C, peptides diluted in serum-PBS (1 mM in 100 µl), or serum-PBS only, were applied and incubated for 4 hours. Bacterial sampling was performed by washing the reaction chambers twice with 250 µl of 10 mM phosphate buffer, pH 7.4, 0.05wt% Triton X-100, supplemented with 0.1% dextran sulfate, added to block peptide activity during sampling (average molecular weight 500 kDa, Sigma-Aldrich, St. Louis, USA). To evaluate the degree of invasive infection, 8 mm skin biopsies were taken after discarding the excess of fluids in the chambers. The biopsies were homogenized, and cfu counts determined.

### Liposome preparation and leakage assay

A liposome model was used in order to study permeabilization of model phospholipid membranes. The liposomes investigated were either zwitterionic (DOPC/cholesterol 60/40 mol/mol) or anionic (DOPE/DOPG 75/25 mol/mol). DOPG (1,2-dioleoyl-*sn*-Glycero-3-phosphoglycerol, monosodium salt), DOPE (1,2-dioleoyl-*sn*-Glycero-3-phoshoetanolamine), and DOPC (1,2-dioleoyl-*sn*-glycero-3-phoshocholine) were all from Avanti Polar Lipids (Alabaster, USA) and of >99% purity, while cholesterol (of >99% purity), was from Sigma-Aldrich (St. Louis, USA). The lipid mixtures were dissolved in chloroform, after which solvent was removed by evaporation under vacuum overnight. Subsequently, 10 mM Tris buffer, pH 7.4 (with or without 150 mM NaCl), was added together with 0.1 M carboxyfluorescein (CF) (Sigma, St. Louis, USA). After hydration, the lipid mixture was subjected to eight freeze-thaw cycles consisting of freezing in liquid nitrogen and heating to 60°C. Unilamellar liposomes of about Ø140 nm were generated by multiple extrusions through polycarbonate filters (pore size 100 nm) mounted in a LipoFast miniextruder (Avestin, Ottawa, Canada) at 22°C. Untrapped CF was removed by two subsequent gel filtrations (Sephadex G-50, GE Healthcare, Uppsala, Sweden) at 22°C, with Tris buffer (with or without 150 mM NaCl) as eluent. CF release from the liposomes was determined by monitoring the emitted fluorescence at 520 nm from a liposome dispersion (10 µM lipid in 10 mM Tris, pH 7.4). An absolute leakage scale was obtained by disrupting the liposomes at the end of each experiment through addition of 0.8 mM Triton X-100 (Sigma-Aldrich, St. Louis, USA). A SPEX-fluorolog 1650 0.22-m double spectrometer (SPEX Industries, Edison, USA) was used for the liposome leakage assay. Measurements were performed in triplicate at 37°C.

### Fluorescence microscopy

For study of membrane permeabilization, the impermeant probe FITC was used. *E. coli* ATCC 25922 bacteria were grown to mid-logarithmic phase in TSB medium, and bacteria were washed and resuspended in 10 mM Tris, pH 7.4, 0.15 M NaCl, with 10 mM glucose, to yield a suspension of 1×10^7^ cfu/ml. 100 µl of the bacterial suspension was incubated with 30 µM of the respective peptides for 2 h at 37°C. Microorganisms were then immobilized on poly (L-lysine)–coated glass slides by incubation for 45 min at 30°C, followed by addition onto the slides of 200 µl of FITC (6 µg/ml) in the appropriate buffers and incubated for 30 min at 30°C. The slides were washed and bacteria fixed by incubation, first on ice for 15 min, then in room temperature for 45 min in 4% paraformaldehyde. The glass slides were subsequently mounted on slides using Prolong Gold antifade reagent mounting medium (Invitrogen, USA). For fluorescence analysis, bacteria were visualized using a Nikon Eclipse TE300 (Nikon, Melville, NY) inverted fluorescence microscope equipped with a Hamamatsu C4742-95 cooled CCD camera (Hamamatsu, Japan) and a Plan Apochromat ×100 objective (Olympus, Orangeburg, NY). Differential interference contrast (Nomarski) imaging was used for visualization of the microbes themselves.

### Statistics

Values are reported as means ± standard deviation of the means. To determine significance, analysis of variance with ANOVA (SigmaStat, SPSS Inc., Chicago, USA), followed by *post hoc* testing using the Holm-Sidak method, was used as indicated in the figure legends, where “n” denotes number of independent experiments. Significance was accepted at p<0.05.

## Results

### Initial survey of antibacterial and hemolytic effects

**Figure 1:**
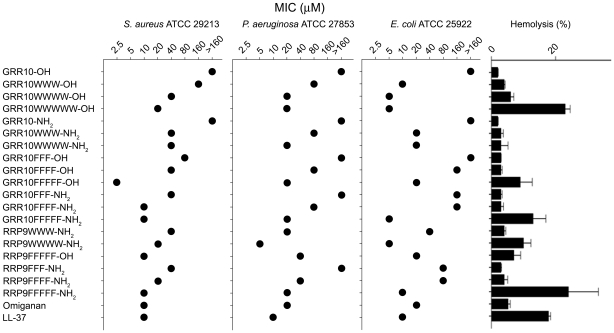
Antibacterial and hemolytic activity of peptides. Minimal inhibitory concentrations (MIC) [60] of the indicated peptide sequences (see Table 1) based on the original sequences GRRPRPRPRP (GRR10) and RRPRPRPRP (RRP9) against various bacterial isolates are shown in the three leftmost diagrams. For comparison, the peptides omiganan and LL-37 are included as well. For hemolysis (rightmost bar diagram), erythrocytes were incubated with the peptides at 60 µM, while 2% Triton X-100 served as positive control. The absorbance of hemoglobin release was measured at 540 nm and is expressed as % of Triton X-100 induced hemolysis. An increased tag-length was associated with low MICs but also increased hemolysis.

A series of W- and F-amino acid tagged peptides, comprising W/F-stretches of 3-5 amino acid residues were used as tags for the template sequences GRRPRPRPRP (GRR10) and RRPRPRPRP (RRP9). Table 1 illustrates the specific sequence and hydrophobicity of the various peptides investigated. As shown in Figure 1, tagging of these template peptides with W- and F-containing amino acid stretches yielded low MICs, particulary for tags containing 4-5 W- or F-residues. For all bacteria, increased tag-length led to increased antimicrobial activities (resulting in lower MIC values). For the peptides studied, C-terminal amidation did not result in significant improvement of activity. The non-tagged peptides showed no hemolysis above that of the negative control (Figure 1). Tagging with 3-4 W/F amino acid residues yielded a slight increase of hemolysis, whereas the longer forms tagged with WWWWW or FFFFF resulted in significantly increased hemolysis. It is of note that the hemolytic activity of those tagged peptides, which showed increased permeabilization of erythrocytes in PBS, was completely abolished in the presence of human citrate-blood (Figure S1).

**Figure 2:**
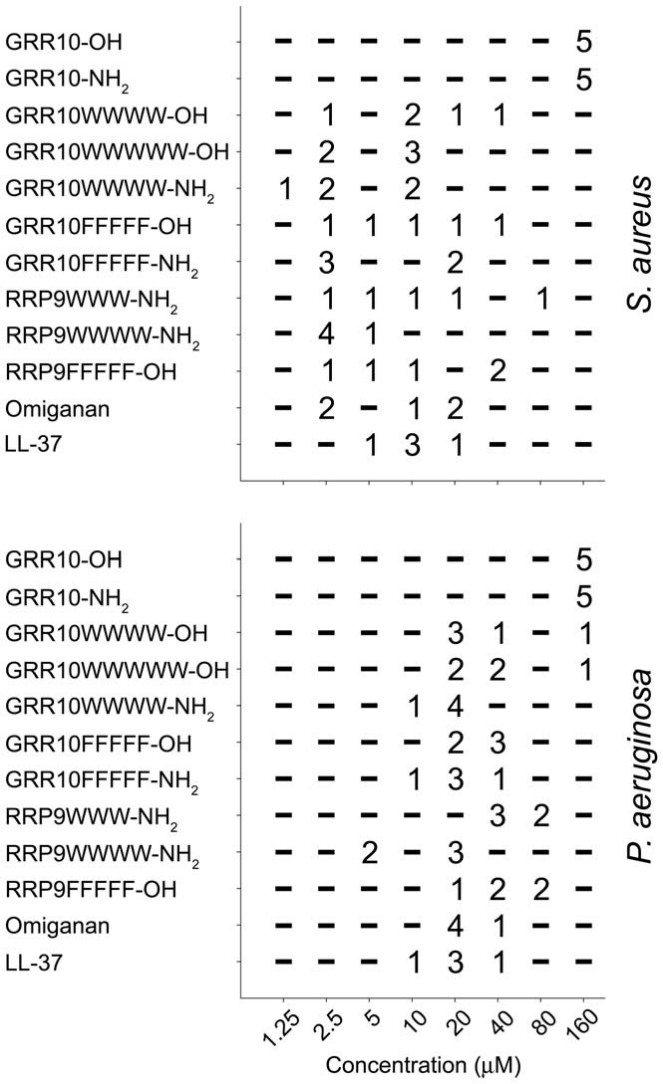
Minimum inhibitory concentrations of indicated peptides against different *S. aureus* and *P. aeruginosa* isolates. Analysis was performed according to NCSLA guidlines in MH-broth. The numbers indicate the number of bacterial isolates presenting MIC breakpoints at the specified concentration range. Some peptides, such as RRP9WWWWW-NH_2_ showed low MIC values against both *S. aureus* and *P. aeruginosa.*

Peptides displaying a low MIC paired with low or moderate increases in hemolysis, thus exhibiting a preferable therapeutic index, were selected for further MIC analyses using various *S. aureus* and *P. aeruginosa* clinical isolates. It was noted that several of the tagged peptides showed MIC values in the range 2.5–40 µM and 10–40 µM for *S. aureus* and *P. aeruginosa*, respectively (Figure 2). The peptide RRP9W4N was particularly active, with MIC values lower than those observed for omiganan and LL-37.

### Peptide toxicity, membrane selectivity, and effects in plasma

**Figure 3:**
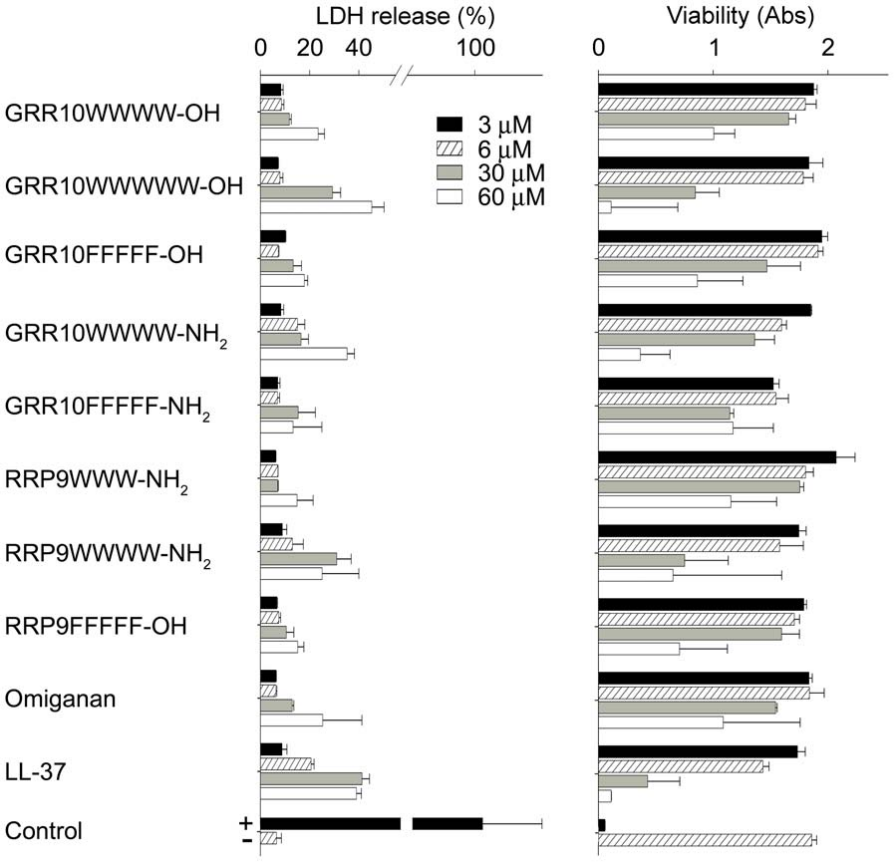
Effect of peptides on mammalian cells. Left panel: Cell permeabilizing effects of the indicated peptides on HaCaT cells, were determined measured by the LDH-based TOX-7 kit. Right panel: The MTT-assay was used to measure viability of HaCaT keratinocytes in the presence of the indicated peptides. The template peptides GRR10 and GRR10-NH2 did not induce any LDH release, nor did they affect viability of the cells (not shown). 1% Triton X-100 (yielding 100% LDH release) (+) and buffer (-) are presented as controls. Overall, a high peptide-mediated LDH-release, particularly at 30-60 µM, was associated with decreased cell viability.

In order to further delineate possible peptide-mediated toxic effects on epithelial cells, dose-response studies, using peptides displaying a low MIC paired with low or moderate increases in hemolysis (Figure 2), were employed using HaCat keratinocytes (Figure 3). Similarly to the results obtained with erythrocytes, tagging increased the permeabilization and concomitantly decreased viability, as measured by LDH and MTT, respectively. It is of note however, that some peptides, e.g., RRP9W4N, showed a relatively low permeabilizing activity at 60 µM, as well as low toxicity as demonstrated by the MTT assay. The permeabilizing activity was concentration dependent, with results for the forms with 3-4 W/F residues being comparable to those obtained for omiganan, and in many cases, significantly lower that that of LL-37 (Figure 3). Hence, considering the obtained MIC values of 2.5–40 µM, the results indicate that the tagged AMPs display considerable selectivity for bacterial membranes.

**Figure 4:**
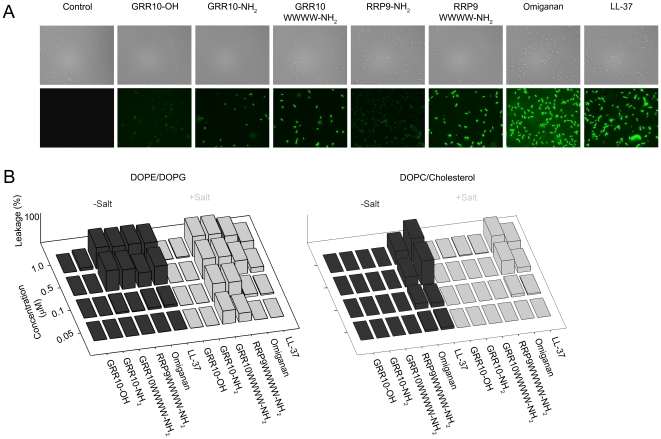
Peptide-mediated permeabilization of bacteria and liposomes. (A) Effects of the indicated peptides on *E. coli*. *E. coli* 25922 was incubated with the indicated peptides (30 µM) after which permeabilization was assessed using the impermeant probe FITC. The upper images in each row are Nomarski Differential Interference Contrast images, while the lower show FITC fluorescence of bacteria. Hydrophobic tagging increases peptide-mediated permeabilization of bacteria at physiological salt strength. (B) Effects of peptides on liposomes in the presence and absence of 0.15 M NaCl (salt). The membrane permeabilizing effect, and resulting release of carboxyfluorescein from liposomes, was recorded by fluorescence spectroscopy. Left and right panel shows anionic DOPE/DOPG (75/25 mol/mol) and zwitterionic DOPC/cholesterol liposomes, respectively. The tagged peptides RRP9W4N and GRR10W4N show a pronounced preferential action on anionic DOPE/DOPG-containing liposomes (mean values are presented, n = 3).

**Figure 5:**
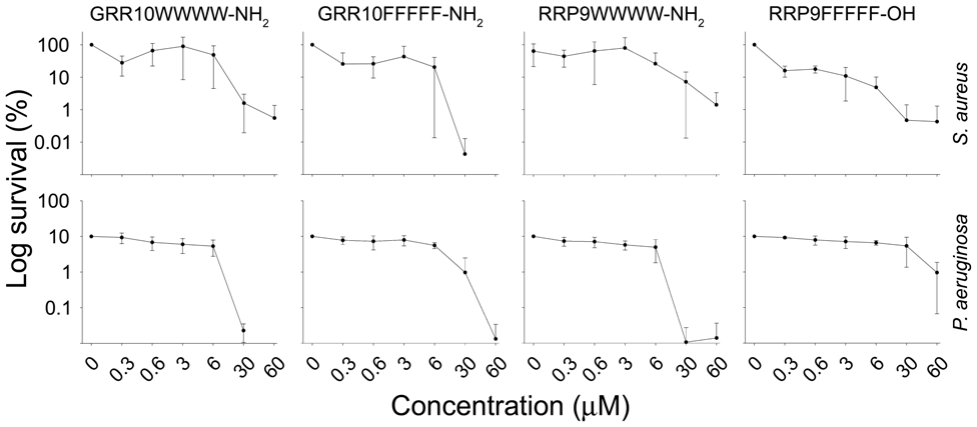
Activities of selected W- and F-tagged peptides at physiological conditions. In viable count assays, *S. aureus* ATCC 29213 and *P. aeruginosa* ATCC 27853 were subjected to the indicated peptides in 10 mM Tris pH 7.4 containing 0.15 M NaCl and 20% human citrate-plasma. Identical buffers without peptide were used as controls. The results with the template peptides GRR10-OH and GRR10-NH_2_ were similar to the controls (not shown).

As can be seen in Figure 4A, and using *E. coli* as model system, the selected W-tagged peptides induced a significant permeabilization of the bacteria when compared with the template sequences. Correspondingly, the tagged peptides were much more potent than the corresponding non-tagged ones in causing membrane rupture of, and leakage from, anionic and bacteria-mimicking DOPE/DOPG liposomes. In contrast, leakage induction was quite limited for DOPC/cholesterol liposomes (mimicking eukaryotic cell membranes) (Figure 4B). Notably, a significantly less selective liposome leakage induction was observed for omiganan and LL-37. Selected peptides with low MICs and a relatively high therapeutic index were further analyzed for antimicrobial activities in the presence of human plasma. As seen in Figure 5, the tagged peptides retained antimicrobial activity at 30–60 µM against both *S. aureus* and *P. aeruginosa*.

### Protease effects on peptides

**Figure 6:**
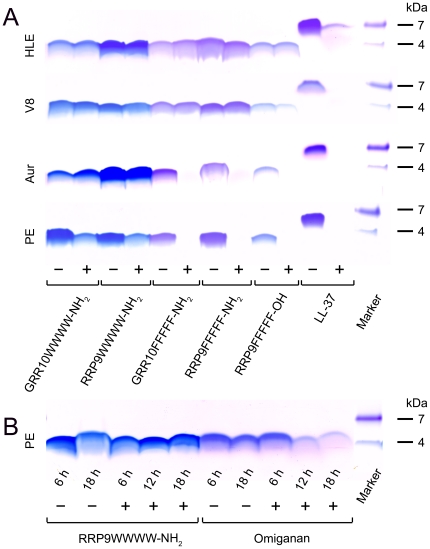
Protease sensitivity of peptides. (A) The indicated W- and F-tagged peptides were incubated with (+) or without (-) human leukocyte elastase (HLE), the *S. aureus* enzymes aureolysin (Aur), V8 proteinase (V8), or *P. aeruginosa* elaslatse (PE) for 4 h at 37°C, and analyzed by SDS-PAGE (16.5% Tris-Tricine gels). (B) As above, peptides were incubated with *P. aeruginosa* for different times up to 18 h and analyzed by SDS-PAGE. Reduced or no staining of peptide indicates partial or complete degradation (peptide fragments are poorly detected). Molecular masses (kDa) are indicated.

We next evaluated the protease stability of the tagged AMPs. As seen in Figure 6A, GRR10WWWW-NH_2_ and RRP9WWWW-NH_2_ were largely unaffected by the proteolytic actions of human neutrophil elastase, as well as the bacterial *P. aeruginosa* elastase and the two *S. aureus* enzymes V8 metalloproteinase and aureolysin. Of note is that the F-tagged forms were degraded by *P. aeruginosa* elastase and staphylococcal aureolysin. In contrast, but in agreement with previous findings, LL-37 was extensively degraded by all these enzymes. The high stability of the W-tagged peptides is illustrated by Figure 6B, demonstrating maintained stability of the peptide RRP9WWWW-NH_2_ after treatment with *P. aeruginosa* elastase. This in contrast to omiganan, which was degraded during extended digestion.

### Activities of peptides in infected blood and skin wound models

**Figure 7:**
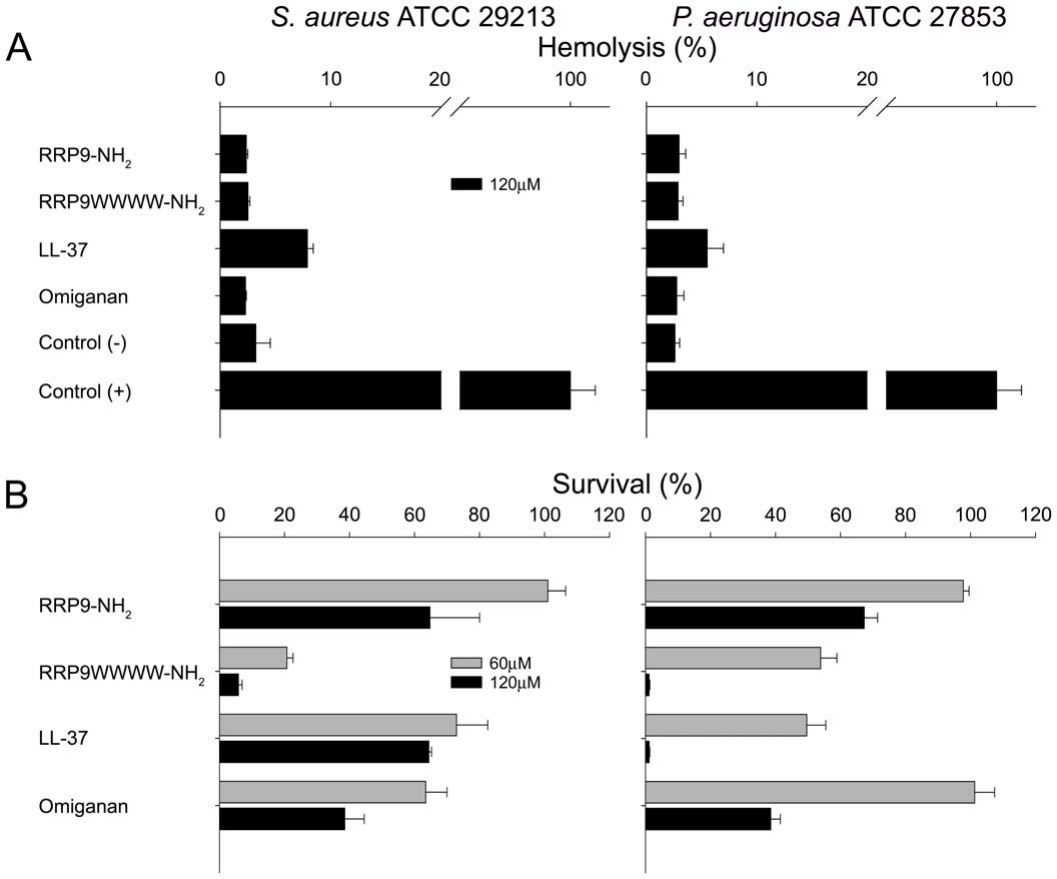
Simultaneous analysis of peptide-mediated hemolysis and antibacterial activity in human blood infected by bacteria. *S. aureus* and *P. aeruginosa* (2×10^8^ cfu/ml) were added to 50% citrate blood, followed by addition of peptide at 60 or 120 µM. (A) Hemolysis in human blood (made 50% in PBS) in presence of the indicated bacteria as well as peptides is presented. Hemolysis was assessed after 1 hour. 1% Triton X-100 (yielding 100% LDH release) (+) and buffer (-) are presented as controls. (B) Using the same material, antibacterial effects (after 1 hour) of the indicated peptides were determined. The number of cfu is presented.

**Figure 8:**
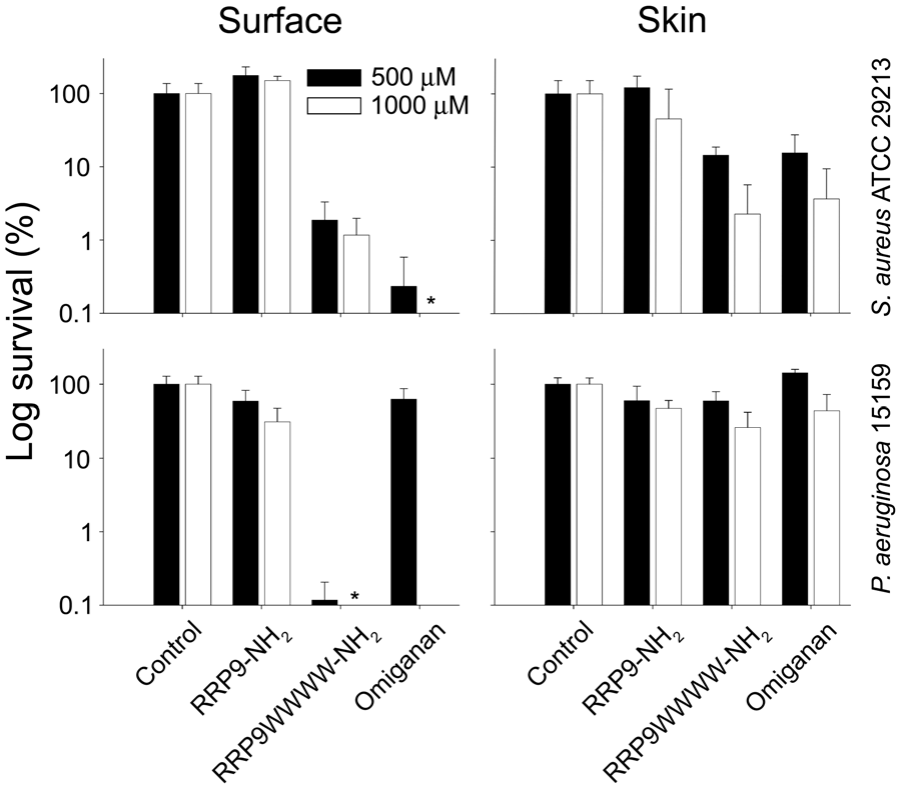
Activities of peptides in an *ex vivo* skin infection model. 6 mm punch biopies were made to pig skin, and the epidermal parts removed, leaving a dermal wound. The wounded area was infected by *P. aeruginosa* 15159 (clinical chronic ulcer isolate) or *S. aureus* ATCC 29213. After an incubation time of 2 hours at 37°C, the peptides RRP9-NH_2_, RRP9WWWW-NH_2_, and omiganan in buffer were applied and incubated for 4 hours (see Material and Methods). Surface-associated bacteria-containing material was collected and CFU determined (surface). To evaluate the degree of invasive infection, skin biopsies were thereafter made, homogenized, and the number of cfu determined (skin) (mean values are presented, n = 3. Note the logarithmic scale on the y-axis). In contrast to the control peptide, RRP9WWWW-NH_2_ reduced the level of bacteria, particularly at the skin and wound surface. (There is a statistically significant difference (P<0.001 two way ANOVA) between the tagged peptide vs. control as well as the native peptide (RRP9NH_2_) at the two doses.).

The stability of the W-tagged peptides, notably RRP9WWWW-NH_2_ against a range of proteases, combined with its potent bactericidal effects, could make this peptide a potential therapeutic candidate. The antimicrobial peptide RRP9WWWW-NH_2_ was therefore added to human blood infected by *S. aureus* or *P. aeruginosa,* and both hemolysis and antibacterial activity was recorded (in the same sample) to further investigate selectivity in a relevant biological context. It was observed that the peptide displayed a striking selectivity, demonstrating almost complete eradication of bacteria added to the blood, with no accompanying hemolysis, at a peptide dose of 120 µM. In contrast, killing of *P. aeruginosa* and *S. aureus* by the peptides LL-37 and omiganan was largely inhibited in this environment, although LL-37 retained activity against *P. aeruginosa* at the highest dose. However, at this level, LL-37 also displayed a significant concomitant hemolysis (Figure 7). Furthermore, in order to study a potential topical antimicrobial effect of RRP9WWWW-NH_2_, a previously established *ex vivo* skin wound infection model was utilized in order to test the efficiency of this tagged peptide. As seen in Figure 8, RRP9WWWW-NH_2_ potently reduced the level of bacteria particularly at the skin and wound surface. It is of note that RRP9WWWW-NH_2_ also reduced deeper bacterial growth, particularly noted for *S. aureus*. At 500 µM, RRP9WWWW-NH_2_ appeared to significantly reduce *P. aeruginosa* to a higher extent than omiganan. As expected, the non-tagged template RRP9N was inactive in all cases. Finally, as presented in Table 2, the peptide RRP9WWWW-NH_2_ showed efficiency against various multiresistant bacterial isolates, including staphylococcal isolates such as MRSA, macrolide resistant Group A streptococci, *E. coli* ESBL, as well as multi-drug resistant *P. aeruginosa.*

## Discussion

Three main findings are presented in this report. First, it shows the applicability of hydrophobic tagging as a means for enhancing antimicrobial potency of ultra-short, highly cationic, and hydrophilic peptide stretches from AMPs, such as the herein described sequences from PRELP. Second, the results indicate that a precise “tuning” of toxicity and proteolytic stability may be achieved by changing tag-length or adding W- or F-amino acid tags, the latter being particularly sensitive to proteolytic inactivation. Third, the optimized peptide RRP9WWWW-NH_2_ retained high antimicrobial potency at physiological conditions, including effects against various multi-drug resistant “superbugs”, demonstrated high selectivity against bacteria in human blood, and showed therapeutic potential in an *ex vivo* model of skin wound infection with *P. aeruginosa* and *S. aureus*.

Although hydrophobic modifications can be designed in a number of ways, including point mutations of individual amino acids or acyl modification, end-tagging by hydrophobic oligoamino acid stretches constitutes an attractive alternative. It allows the primary AMP sequence, such as the PRELP-derived sequence reported here, to be retained, while an efficient, but selective, membrane anchoring is achieved. W-tagging also does not affect proteolytic stability of the tagged AMPs detrimentally, a factor of importance for bactericidal potency on *S. aureus* and *P. aeruginosa*, as well as other bacteria secreting AMP-degrading proteases [62]. The present results also indicate that utilization of F tags may be an efficient means of reducing proteolytic stability of the AMP in a controlled way, of potential interest in situations where a rather limited and time-dependent antimicrobial effect is preferred, leading to inactivation of the administered AMP and generation of the completely endogenous template sequence in a prodrug context.

As mentioned above, AMPs may mediate bacterial killing by both membrane disruptive and non-disruptive ways. Concerning membrane disruptive effects, some peptides, such as melittin, alamethicin, magainin 2 and gramicidin A may form transmembrane structures [11], [63], [64], [65]. Disordered and highly charged peptides, including the ones studied here, disrupt membranes by other mechanisms, involving generation of negative curvature strain, membrane thinning, or local packing defects associated with peptide localization within, or close to, the phospholipid polar headgroup region [11], [30], [66], [67], [68], [69]. In the latter cases, membrane defect formation increases with the amount of peptide bound to the lipid membrane, hence high peptide adsorption at the membrane promotes AMP potency [30], [66], [67], [68]. Due to potential lytic properties of AMPs against bacterial as well as mammalian membranes, one of the challenges in designing new peptides relies on developing AMPs with high specificity against bacterial cells, i.e., a high therapeutic index. The finding that RRP9WWWW-NH_2_ displayed no lytic activities against mammalian cells in blood, while simultaneously effectively killing bacteria (Figure 7), suggest a quite remarkable dissociation between antimicrobial and antieukaryotic activities. Taken together, these data, combined with results from the other analyses on bacteria, erythrocytes, keratinocytes, and liposomes, clearly indicate that the W-tagged peptide displays a high selectivity against negatively charged bacterial membranes. Likely, the underlying mechanism for the latter selectivity depends on the fact that bulky groups such as W and F require substantial area expansion for their incorporation in phospholipid membranes [70], and therefore tag insertion into zwitterionic eukaryotic membranes containing strongly membrane-condensing cholesterol becomes an energetically costly process, whereas the driving force to peptide binding is higher, and the energetic penalty for peptide incorporation in the phospholipid membrane is lower, for highly negatively charged and cholesterol-void bacterial membranes.

From a clinical perspective, alternatives to antibiotics and antiseptics are highly needed. A number of different antimicrobial strategies may be deployed for the prevention or treatment of infected wounds. Concerning topical antimicrobials, various antiseptics have long and commonly been used on wounds to prevent or treat infection. Several antiseptic categories exist, including alcohols (ethanol), anilides (triclocarban), biguanides (chlorhexidine), bisphenols (triclosan), chlorine compounds, iodine compounds, silver compounds, peroxygens, and quaternary ammonium compounds [71]. Various antimicrobial agents are used for both intact skin and wounds, although concerns are raised based upon effects on human cells and wound healing, such as those observed for silver [34], [72]. Furthermore, although the multifaceted effect of silver carries a low risk of resistance, studies in burn wounds have shown that bacteria, and in particular *P. aeruginosa* may become resistant to silver compounds (such as silver sulfadiazine and silver nitrate) [34]. As mentioned above, various AMPs may constitute new therapeutic alternatives for topical use. Attractive features of the tagged AMPs presented in this study include a broad-spectrum activity against multiresistant bacteria and efficiency in *ex vivo* wound infection models, a high selectivity and low toxicity, and the possibility of precise “tuning” of both effect and proteolytic stability. Clearly, further investigations involving *in vivo* animal models, toxicological analyses, as well as clinical studies, are mandated in order to further explore the potential of these AMPs as novel antiinfectives.

## Supporting Information

Figure S1 **Hemolytic activity of peptides in human blood.** Citrate-blood was diluted (1∶1) with PBS. The cells were then incubated with end-over-end rotation for 1 h at 37°C in the presence of the indicated peptides (at 60 µM). 2% Triton X-100 (Sigma-Aldrich) served as positive control. The samples were then centrifuged at 800 g for 10 min. The absorbance of hemoglobin release was measured at λ 540 nm and is in the plot expressed as % of TritonX-100 induced hemolysis.
(PDF)
