# Supplementary material for: Highly Selective End-Tagged Antimicrobial Peptides Derived from PRELP
Source: PLoS One. 2011 Jan 27;6(1):e16400. doi: 10.1371/journal.pone.0016400 (PMC3029338; doi:10.1371/journal.pone.0016400)
Supplement: Figure S1 — Hemolytic activity of peptides in human blood. Citrate-blood was diluted (1∶1) with PBS. The cells were then incubated with end-over-end rotation for 1 h at 37°C in the presence of the indicated peptides (at 60 µM). 2% Triton X-100 (Sigma-Aldrich) served as positive control. The samples were then centrifuged at 800 g for 10 min. The absorbance of hemoglobin release was measured at λ 540 nm and is in the plot expressed as % of TritonX-100 induced hemolysis. (PDF) [file pone.0016400.s001.pdf]

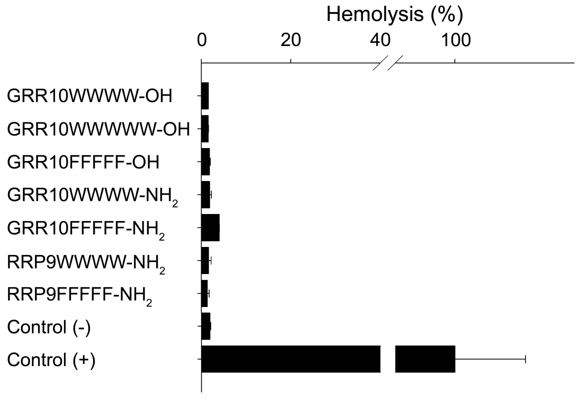
Hemolysis (%)
0
20
40
100
GRR10WWWW-OH
GRR10WWWWW-OH
GRR10FFFFF-OH
GRR10WWWW-NH2
GRR10FFFFF-NH2
RRP9WWWW-NH2
RRP9FFFFF-NH2
Control (-)
Control (+)
